# The application of unmanned aerial vehicle oblique photography technology in online tourism design

**DOI:** 10.1371/journal.pone.0289653

**Published:** 2023-09-07

**Authors:** Mengyi Lin, Zhaoyang Meng, Caisheng Luo, Yingjiao Chen

**Affiliations:** Fujian Jiangxia University, Fuzhou, Fujiang, China; University of Malta, MALTA

## Abstract

Tilt photography of unmanned aerial vehicles (UAVs) is widely used in urban management, cadastral mapping, disaster monitoring and other fields, but there are few innovative applications in online tourism. In this study, empirical design and usability testing methods were adopted, advanced positioning technology and Cesium engine were used to build a 3D real online travel application based on tilt photography, and the interactive function was realized through JavaScript language dynamic interactive function script. Meanwhile, combined with the questionnaire survey, the mobile application Rating Scale (MARS) was used to evaluate the function and quality of the application. The results show that engagement, functionality and aesthetics have no significant influence on user perception. Information quality and subjective quality of application have significant positive influence on user perception, and the influence of information quality is more significant. This study extends the application of oblique photography in tourism and provides experience for the development of tourism digitization.

## 1. Introduction

In recent years, the application of remote sensing mapping technology based on UAV photography has made great progress, which has important application value in scientific research, especially in the fields of ecological environment monitoring [1–4] and climate change [5, 6] Oblique photography is a remote sensing surveying and mapping technology developed in recent years in the field of international surveying. It overcomes the previous limitation that orthophotos can only be shot from a vertical angle, and can quickly draw a 3D model through professional data processing software to present the real scene. Oblique photography mainly relies on UAV images for high-precision 3D models, which is featured with flexibility, high efficiency, low cost, multiple angles, large scale, high definition, small amount of data and easy publishing through Internet [7–9]. It can display the real physical world in all directions and with all elements, and due to the absolute advantages of high precision, high efficiency, high realism and low cost, it has become an important data source of 3D GIS [10]. In the past, oblique photography has been widely used in various fields such as rural land surveying and mapping [11–13], planning and design [14, 15], river and lake management [16], emergency command [17], homeland security [18], urban management [8, 19–21] and disaster monitoring [22–25]. In recent years, with the rapid development of intelligent tourism and information transmission technology, oblique photography technology has gradually played a major role in the field of smart tourism. Some scholars have discussed the application of oblique photography technology in smart city construction and smart tourism, but most of them focused on the layout of oblique photography 3D models in smart city construction, VR tours in smart tourism [14], and tourism resource development and protection [26, 27]. At present, in the dynamic management and application practice of smart parks, it is mainly adopted to the back-end management platform of the government or marketing service agencies, and a few discussions have been applied to online tourism innovation and marketing services of tourist destinations. The specific application of smart tourism is not discussed in the current communication environment and marketing environment, and based on the management optimization that smart tourism can get and the effective promotion of sustainable development.

With the rapid development of digital twin technology, tourist destinations apply digital technology to inform tourists about the destination (especially the services that can be provided) in a targeted manner, which can provide references for tourists to choose travel destinations and make their travel itineraries. In recent years, social media have also become a very valuable data source for tourism analysis because of its availability and the significant online presence of tourists on social networks [28].

Therefore, it is necessary to analyze the preferences of tourists through the data of online trajectories and behavioral habits used by tourists, so as to provide decision-making basis for the optimization of tourists’ service design. The purpose of this study is to realize the data precipitation of tourist destinations, including the data summary generated by scenic spots, hotels, tourists, suppliers, traffic data and word-of-mouth comments, through the combination of tilt photography technology and the function of social media online, so as to provide the research basis for the transformation of destination tourism data. It can be a key node in the online tourism chain. Provide the basis for all decisions. The greatest contribution of this study is to reconstruct the interaction mode between tourist destinations and relevant stakeholders by combining tilt photography technology, and to provide theoretical and applied basis for the application construction of remote sensing technology in the field of tourism service platform. The study mainly consists of three parts:

1. Practical design of tilt photography equipped with wechat mini program. The online tourism service application demo (East China Sea Wonderland mini program) combined with social media is developed. Based on the tilt photography technology of UAV, the application scenario of real scene imaging of tourist destinations is applied to solve the precipitation of basic tourism data, improve the credibility and dissemination of destination information, and make the relevant information of tourist destinations more convincing to meet the needs of potential tourists.
2. Usability test of the East China Sea Wonderland mini program. First of all, through the interview method and observation method, to test whether the interactive system of small program is easy to use, the operation interface and the use perception of the panoramic navigation interface. At the same time, the snowball sampling method was adopted to conduct a questionnaire survey with the mobile application Rating Scale (MARS). It was assumed that participation, functionality, aesthetics, information quality and subjective quality all played an active role in the perceived impact. Then spss was used for statistical analysis to evaluate the function and quality of the demo, and explore whether the relevant assumptions were valid and their influence and relationship.
3. Discuss the research results and offer suggestions on the future improvement direction and operation of the online travel application of slanting photography combined with mobile social networking.

## 2. Combination of oblique photography and social media in tourist destinations

### 2.1. Limitations of social media in tourism marketing

Social media is a platform of content production and sharing based on user relationships on the Internet. At present, many marketing contents are published and maintained based on social media (such as WeChat official accounts). Limited by personal social boundaries, the publication of information on social media is open to a certain extent, which can trigger spontaneous discussions within the corresponding social circles, produce hot spots and realize targeted communication, thereby playing an important role in tourism activities among both tourists and suppliers. Past research and studies have shown that travel information disseminated and searched for on social media has a significant impact on tourists’ decision-making and behavior [29]. Social media is one of the main sources of tourist information [30], they collect information about destinations, transportation and accommodation deals, compare prices and services, and search for related photos and videos of destinations [31, 32]. Social media have become one of the primary data sources for the analysis of tourist activities in the last decade, due to the enormous amount of heterogeneous information that they provide (messages, pictures, videos, opinions, ratings, etc.) [28]. However, from the perspective of tourism marketing, the use of social media for communication and dissemination is featured with the following limitations.

1. The information released by social media, whether by institutions or individuals, is endowed with a certain degree of subjectivity, which cannot fully demonstrate the true situation of tourism resources. Some biased output is even likely to have an impact on the evaluation of tourist destinations by consumers and potential tourists.
2. The current channels of content interaction of social media is mainly text, voice and pictures. Even though short videos are popular at present, all the above channels lack a sense of immersion in terms of content presentation. For the so-called experiential marketing and substitution marketing in the current marketing field, they still lack a certain degree of attractiveness, and are neither direct nor deep enough in terms of marketing, tackling the pain points and stimulating the impulse to consume.
3. Currently, the information on the Internet is dazzling. Due to the influence of some over-packaging and false information, the public’s trust in the information obtained in social media has declined. At present, if the lagging problem of social media information can be solved, and real-time interaction and real scene presentation can be formed between social media and consumers, tourists’ perception of trust in tourist destinations is bound to be improved.

### 2.2. Complementation of oblique photography to tourism marketing

Oblique photography is a technology that can use imaging technology to present a certain location in real life. It can display the all-round scenery, style, culture, transportation, etc. of the tourist destination, and be carried on social media, of which the real-time interaction function can realize real-time communication and sharing of the scenery in tourism destinations. The combination of oblique photography and social media allows tourism operators, tourists, potential customers, and information searchers to conduct targeted information exchanges on a platform with real-life display, and timely solve problems caused by unclear information or simple text and images. Potential customers and information searchers can search and inquire according to their own needs. Tourists can immediately share the actual feelings and travel experience at every geographic point of the tourist destination through the adoption of oblique photography. Combining the real situation of the tourist destination with the actual feelings of tourists, it breaks through the problem in relying solely on social media and not being able to achieve situation immersion. Tourism operators can present the tourist destination in a more realistic way based on the 3D modeling of the real scene, while effectively interacting with immediate tourists and potential tourists about the destination. Meanwhile, based on the results of 3D images shot through oblique photography and through photogrammetry from multiple angles and large-scale mapping, corresponding geographic information and surrounding conditions about the tourist destination can be provided. Combined with software such as Context Capture, Cesium and Street Factory and through the real 3D modeling of the tourist destination, it can truly reflect the surrounding environment of the tourist destination and the attributes of buildings such as appearance, location and height. In the later data application, ArcGIS software is used for data vectorization, and the roads, buildings, landscapes and other objects of the destination are observed. Through advanced positioning technology, accurate geographic, humanistic and marketing information are embedded to present 3D real-life maps and road books of the destination, which can greatly expand the application field of basic data of tourist destinations. While forming accurate data on tourist destinations, it will also provide accurate and clear data for subsequent maintenance and repair of the destination. Therefore, oblique photography ensures the panoramic presentation of tourist destinations and solves the problem in the credibility of tourist destination marketing, which explains the purpose of this research combining oblique photography technology with social media development.

## 3. Practical design and presentation

### 3.1. Combination of oblique photography with WeChat mini program in the scenic spot Tokai Wonderland

This paper chooses China’s WeChat mini program, a social media APP, as the case study object to design empirical research. WeChat is a free APP that provides instant messaging service for smart terminals, it was launched on January 21, 2011 by the Chinese company Tencent. It is an open service platform integrating social network communication, mobile payment, e-commerce and public services. WeChat mini program was released on January 9, 2017, and it is so convenient that it can be used without downloading or installing. Users only need to scan a certain QR code or search the name of the mini program in WeChat, and it is connected to dozens of WeChat access ports. According to the “2019 WeChat Data Report” released by WeChat official in January 2020, WeChat has more than 1.1 billion monthly active users globally and 300 million active users of mini programs (Tencent, 2020), which is widely used in China. The development tool of WeChat mini program can develop different functional modules according to the individual needs of users. Meanwhile, users can easily query and switch between the company official account and the mini program, which can not only optimize users’ experience, but also easily transfer mini program users into brand followers, strengthening the connection between destination brands and consumers. Therefore, this research applies oblique photography to such mature, convenient and open service platform, and applies it to online travel services, which is convenient for developers and reduces the capital investment of operators. The new way to connect users and services through WeChat mini program provides the best carrier for online travel data collection of tourist destinations, meanwhile offering convenience to users, enhancing the connection between tourist destinations and consumers, and creating a sustainable opportunity for follow-up services.

**Fig 1:**
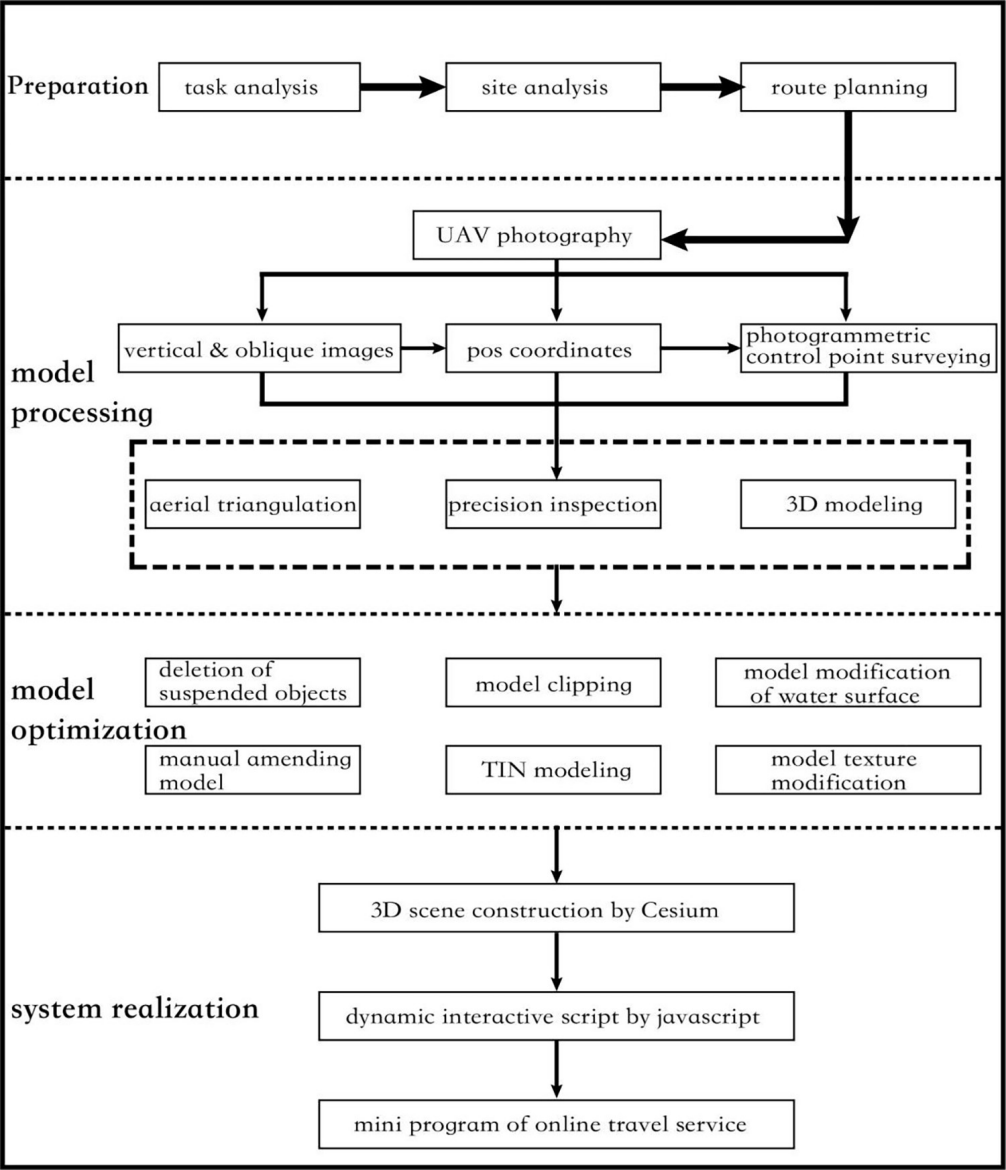
The design flowchart of combining oblique photography 3D modeling with WeChat mini program in the scenic spot Tokai Wonderland.

This research takes Tokai Wonderland in Pingtan Comprehensive Experimental Zone of China as a practical case study object. The scenic spot Tokai Wonderland is located in the east of Fujian Province, adjacent to the eastern sea area, with an area of 7.5 square kilometers. Due to its geological structure, long-term seawater erosion and sand erosion, it has developed into a magnificent, mysterious and complex marine abrasion landform. In the early stage of this research, field investigations were carried out many times from 2018 to 2019 to test and the feasibility of UAV flight and inspect the acquisition layout of control points in Tokai Wonderland, and the modeling was conducted afterwards. The application design is shown in Fig 1.

The first three steps are basically common in the industry. In system implementation, the software is selected according to the special attributes of different industries to realize scene optimization and interactive script design. The specific steps are as follows.

1. Oblique images are collected through UAV photography. Whether the flight directions of the side-view images of close flight routes are consistent needs checking, and aerial triangulation is conducted after the confirmation of the position of image placement and camera parameters.
2. After confirming that the image data is correct, aerial triangulation is conducted. A small number of field control points are encrypted to obtain accurate external position elements, and the connection points are found through dense image matching of multiple points to construct 3D TIN. For zones with a large number of data, separate data zone processing is required. In this research, adaptive zonation is resorted to, which can quickly adapt to the computer’s RAM. According to the current performance of the computer, a referential range is given, and the target RAM is set within the range, so as to successfully carry out the 3D reconstruction.
3. The corresponding optimal texture information is selected according to the angle between the normal line equation of each triangular in the 3D TIN and the 2D image to conduct automatic texture mapping.
4. 3D scene model in OBJ format is output and imported into 3D System Warp for scene optimization, in which deletion of suspended objects, model clipping, model modification can be operated.
5. The optimized model is imported into Context Capture for scene repair and exported in Cesium 3dtiles format to Cesium engine, and dynamic interactive script is produced through javascript to realize real-time driving of Tokai Wonderland and nearby scenes and the online travel function of mini program in user interface.

In the application of the mini program, field scanning is carried out to restore the complex 3D scene of the scenic spot through UAV oblique photography, and then the corresponding web page is constructed and adjusted to present the real 3D map of the Tokai Wonderland, which is used in combination with the GPS positioning technology to realize the interest buoy setting within the destination. In addition, and the panorama map drawn through oblique photography can be zoomed in, zoomed out and rotated from multiple angles, which further optimizes the experience of using the mini program. Meanwhile, the demand on the combination of oblique photography with social media is met through adding many other modules.

### 3.2. Construction and application of the Tokai Wonderland mini program

In this research, two management and operation backgrounds were developed to ensure the normal use of Tokai Wonderland mini program. In the background management interface of the mini program, the modules include order management, WeChat membership, scenic spot management, scenic spot list, specialty sale, road book management, lottery settings and system settings. The display of 3D real scene includes presentation center, sand table center, different scenes, point of interest, area of interest, visit track and user center through the independent background module of 3D real scene management. The 3D real scene produced through oblique photography in the mini program is interactively presented through the web link of panoramic map.

The road book includes the hot spot numbers, pictures, titles, routes, instructions, ranking, status and details of the tourist destination. The detailed setting interface includes common content typesetting functions such as setting the font size, color and paragraph, and inserting audio, pictures and map, etc. The order management interface of the background management system of Tokai Wonderland, on which the specialties of the tourist destination are classified into cultural and innovative gifts and local food. The interface of specialties is equipped with general operation functions of e-commerce, including product information, keywords, prices, status and product detail pages. The interface order management contains order number, order time, unit price, quantity, customer information and logistic information.

In the point of interest and view point management interface of 3d real scene background management system. Users can add buoys of their points of interest in the 3D interface of the tourist destination. The system will automatically calculate the latitude and longitude of the point, and insert different types of scene such as scenic spots, catering, accommodation, buildings and public facilities in order to provide users with visit route and enable the dissemination of popular scenic spots in the destination. In terms of the flight track and scene list of 3D real scene background management, the track can be customized into track browsing and flight guide route, and it can realize the track playback of multiple addresses among different scenic spots, providing judgment on the overview of guide route for tourists in advance. The scene list is the control center of all scenic spots in the whole 3D real scene background, which includes the latitude, longitude, height, offset, horizont, direction, tilt and roll of the scenic area, corresponding to the list of scenic spots in the management background of Tokai Wonderland mini program.

In the operation interface of 3d real scene background management system. The points of interest are added and managed through the operation interface of point of interest list. The function interface displays operation options such as name, scene, latitude and longitude, type of scene and brief introduction. The function of brief introduction is similar to that of the setting interface of mini program details, and also includes the commonly used functions such as content typesetting, settings of font size, color, and paragraph, and audio, picture and map inserting. The operation of the functional modules in the two backgrounds is relatively intuitive and clear, and does not require too much technical or professional knowledge, which reduces the requirements for professional talents in the background operation of scenic spots.

## 4. Usability test of the Tokai Wonderland mini program

### 4.1. East China Sea Wonderland small program usability test method

In order to verify the usability of combining WeChat mini program with oblique photography, this research tests and evaluates the prototype of the Tokai Wonderland mini program. A combination of qualitative and quantitative evaluation is resorted to, as well as observation, user interview and questionnaire survey, which are common methods of user research of interaction design and usability testing to measure the usability from multiple perspectives such as usability, simplicity, pleasure, etc [33, 34]. Therefore, this research adopts observation, user interview and questionnaire survey for user testing of the mini program, among which observation and user interview were carried out simultaneously, and 12 mini program users were invited for observation and face-to-face interviews. To ensure the authenticity of the feedback received, all participants have provided verbal consent to participate in this usability testing research and engage in extensive contact and interaction.The main purpose of the observation is to verify whether the interactive system of the Tokai Wonderland mini program is easy to use, users’ feelings about operating on different interfaces and their usage of the panoramic guide map. During the interview, we constantly communicated with the users, and the questions are as follows.

1. Is the Tokai Wonderland mini program easy to understand?
2. Are there any jump pages or information difficult to understand?
3. How does it feel to operate the panorama guide map made through oblique photography? Are you interested in that?
4. Will you share this mini program with friends?
5. Please give your suggestions and expectations for the future improvement of Tokai Wonderland mini program.

A total of 12 users were invited in this research, 6 man and 6 women. Taking into account the use of smart phones, the age group of the participating users ranged from 20 to 60 years old. During the interactive use of the interface, five users hesitated to interact. Among them two could not find the button to enter the panoramic map interface, and managed to enter after we offered assistance. Two hesitated when returning to the list of scenic spots after entering a certain one, instead they quit the mini program and re-entered later. After entering the panoramic guide interface, one hesitated with gestures while viewing the 3D panoramic map. All the 12 users showed willingness to share the mini program to those in need and expressed interest in panoramic guide. Some were even curious about the panoramic guide technology (see as Table 1).

**Table 1:** Record of observation and user interview.

No.	Gender	Age	Hesitation	When using the mini program	Recommendation	Suggestions and feedback
1	Woman	70’s generation	No	Rather smoothly	Yes	Enrich the information
2	Woman	85’s generation	No	Rather smoothly	Yes	Add prompt of returning to the scenic spot list
3	Man	80’s generation	Yes	Unable to find the button to enter the panoramic map	Yes	Add prompt of entering the 3D panoramic map
4	Woman	90’s generation	No	Rather smoothly	Yes	Modernize the interface design
5	Man	60’s generation	No	Rather smoothly	Yes	Enrich the information on the detail page
6	Man	00’s generation	No	Rather smoothly	Yes	Enrich the panoramic map
7	Man	90’s generation	Yes	Unable to find the button to enter the panoramic map	Yes	Add prompt of entering the 3D panoramic map
8	Woman	95’s generation	No	Rather smoothly	Yes	Add panoramic navigation
9	Man	70’s generation	Yes	Had problems in returning to the scenic spot list	Yes	Add prompt of returning to the scenic spot list
10	Man	65’s generation	No	Rather smoothly	Yes	Enrich the information on the detail page
11	Woman	00’s generation	No	Rather smoothly	Yes	Enrich the information on the detail page
12	Woman	65’s generation	Yes	Hesitated with gestures while viewing the 3D panoramic map	Yes	Add prompt of gestures

In terms of suggestions and expectations, most of the 12 users hoped to enrich the information on the detail page, followed by adding the homepage prompt for returning to the scene spot list, adding the function of panoramic navigation, modernizing the interface design to be in line with the popular style that young people prefer today, and increasing gesture prompts.

The questionnaire survey employed the revised version of the Mobile Application Rating Scale (MARS) which was used to assess the quality of mobile health APPs. MARS is considered a standardized criterion for evaluating the quality of a web or APP extracted from 372 evaluations in 25 published papers, meeting minutes and web resources by scholars including Stoyanov. Five categories of criteria were finalized including four objective quality scales, namely engagement, functionality, aesthetics and information quality, and a subjective quality scale (see as Table 2). In addition, items that could be adjusted and used to evaluate the perceived impact of a specific APP on changes in the users’ knowledge, attitudes and intentions, and the likelihood of actual changes brought to the target behavior were added. Excellent internal consistency (Cronbach alpha = .90) and high internal consistency for subscales (Cronbach alpha = .80-.89, median 0.85) were shown [35]. Since its creation, MARS has been used to assess many APPs of different nature, such as the cognitive performance assessment for smart watches [36], physical exercise [37] and smoking cessation [38]. At present, MARS is a popular rating scale for mobile APPs. Since MARS was originally a method for evaluating the quality of mobile health APPs, this research deletes the "inapplicable" sub-items and revises some items in the original version of MARS. A total of 26 questionnaire items were finally generated. The 5-level Likert scale ranging from 1 ("poor") to 5 ("excellent") was employed, and word explanations were added to the scale to ensure that the respondents could understand. Based on the MARS revision, the following hypotheses are proposed:

H1 participation positively affects perception.

H2 function positively influences perception.

H3 aesthetics positively affects perception.

H4 information quality positively affects perception.

H5 application subjective quality positively affects perception.

**Table 2:** The original version of MARS and sub-items.

Scale	Sub-items
engagement	1.entertainment, 2. attention, 3. customization, 4. interactiveness, 5. target group
functionality	6. performance, 7. simplicity, 8. navigation, 9. gesture design
aesthetics	10. layout, 11. graphics, 12. visual attractiveness: how does the APP look?
information quality	13. accuracy of instructions, 14. objectives, 15. information quality, 16. information volume, 17. visual information, 18. reputation, 19. evidence base
subjective quality	20. Would you recommend this APP? 21. How many times do you think you will use this APP? 22. Will you pay for this APP? 23. How many stars will you rate for this APP?

### 4.2. Test results of usability

This research adopts the snowball sampling method to share the WeChat mini program peer to peer and invite respondents to use the Tokai Wonderland mini program, and then an online questionnaire was sent to them to complete. Later in the same way, the respondents were asked to transfer the mini program peer to peer to those who belonged to the target population of this research in order to collect samples, and red packets were added in the questionnaire to encourage receivers to fill in the questionnaire objectively and fairly. At last, a total of 234 questionnaires of the mini program user rating scale were collected, among which 218 valid samples remained after 16 invalid were deleted. Statistical analysis was then performed through SPSS. As shown in Table 3, there were 91 man (41.7%) and 127 women (58.3%), the gender ratio met the requirement. Respondents aging from 26 to 30 years old accounted for the largest proportion 28%, followed by those of 10 to 25 years old, accounting for 58%. In terms of education level, respondents with bachelor degree accounted for 46.8%. The frequencies of whether resident in the Pingtan Comprehensive Experimental Zone were roughly the same, so the samples were randomly chosen.

**Table 3:** Basic information of the samples.

		Frequency	Percentage
Gender	Man	91	41.7
	Woman	127	58.3
	Total number	218	100
Age	Under 18	4	1.8
	18–25	59	27.1
	26–30	61	28
	31–40	58	26.6
	41–50	26	11.9
	51–60	10	4.6
Education level	Primary school	2	0.9
	Middle high school	24	11
	High school	18	8.3
	College	30	13.8
	Bachelor degree	102	46.8
	Master’s degree and above	42	19.3
Whether resident in the Pingtan Comprehensive Experimental Zone	Yes	108	49.5
	No	110	50.5
Total number		218	100

To further analyze the reliability of the questionnaire, the internal consistency coefficient (Cronbach’s coefficient) was employed to test the reliability of the data. Usually the coefficient value is between 0 and 1. In general, the value greater than 0.8 indicates excellent internal consistency, between 0.6 and 0.8 good internal consistency, and below 0.6 poor internal consistency. It can be seen from Table 4 that the corrected item-total correlation value of each item in the questionnaire is greater than 0.3, indicating that most of the items in this questionnaire have a strong correlation with the total item, and the questionnaire design is featured with a degree of discrimination. The Cronbach’s Alpha of each dimension are 0.852, 0.847, 0.838, 0.856, 0.837 and 0.939, which are all greater than 0.8. The total Cronbach’s Alpha of the questionnaire is 0.961, which is greater than 0.9, indicating that the questionnaire has good internal consistency with good stability and reliability. The questionnaire design is reasonable and feasible. The deleted Cronbach’s Alpha of each item is greater than 0.8, indicating that the questionnaire is highly reliable (Table 4).

**Table 4:** Results of reliability analysis.

		Corrected item-total correlation	Deleted Cronbach’s Alpha of each item	Cronbach’s Alpha
Engagement	1.entertainment	0.656	0.827	0.852
	2.attention	0.697	0.820	
	3.customization	0.705	0.817	
	4.interactiveness	0.733	0.806	
	5.target group	0.616	0.836	
Functionality	6. performance	0.631	0.837	0.847
	7. simplicity	0.756	0.773	
	8. navigation	0.724	0.789	
	9. gesture design	0.656	0.821	
Aesthetics	10.layout	0.674	0.805	0.838
	11.graphics	0.724	0.752	
	12.visual attractiveness	0.715	0.769	
Information quality	13.information quality	0.684	0.822	0.856
	14.information volume	0.692	0.822	
	15.visual information	0.712	0.811	
	16.credibility of the source	0.714	0.811	
Subjective quality	17.subjective quality 1	0.675	0.792	0.837
	18.subjective quality 2	0.722	0.772	
	19.subjective quality 3	0.653	0.827	
	20.subjective quality 4	0.709	0.791	
Perceived impact	21.consciousness	0.863	0.922	0.939
	22.knowledge	0.819	0.927	
	23.attitude	0.823	0.927	
	24.intention to change	0.842	0.924	
	25.ask for help	0.768	0.933	
	26.behavior change	0.790	0.931	
Total value			0.961	

From the results of validity analysis in Table 5, it can be seen that the KMO values of each dimension of the questionnaire are 0.814, 0.795, 0.723, 0.827, 0.806 and 0.926, all of which are greater than 0.7. The sig value of approximate chi-square in Bartlett’s test of sphericity of each dimension in Table 5 is close to 0 and less than 0.05, indicating that the scale validity is good.

**Table 5:** Results of validity analysis.

KMO and Bartlett’s test							
Dimension		Engagement	Functionality	Aesthetics	Information quality	Subjective quality	Perceived impact
Kaiser-Meyer-Olkin test with enough samples		0.814	0.795	0.723	0.827	0.806	0.926
Bartlett’s test of sphericity	Approximate chi-square	492.557	380.964	263.638	371.327	371.794	1071.415
	df	10	6	3	6	6	15
	Sig.	0.000	0.000	0.000	0.000	0.000	0.000

It can be seen from Table 6 that the mean of each dimension of the questionnaire is between 3.17 and 3.96, all greater than 3. The dimension with the lowest mean is “subjective quality”, and the largest “perceived impact”.

**Table 6:** Statistical results.

Statistics					
	N	Min	Max	Average	Standard deviation
Engagement	218	1.20	5.00	3.546	.823
Functionality	218	1.25	5.00	3.881	.762
Aesthetics	218	1.67	5.00	3.788	.827
Information quality	218	1.75	5.00	3.936	.731
Subjective quality	218	1.25	5.00	3.171	.945
Perceived impact	218	1.83	5.00	3.956	.783
valid N (list state)	218				

Respondents evaluated the dimension "engagement" by answering questions such as whether the mini program is fun, whether it is attractive, whether it allows for customization, whether it is interactive, and whether it suits the target group. The mean score on this dimension is 3.546 (standard deviation = 0.823).

Functionality is measured based on the user feedback on the accuracy and operating speed of the mini program, the simplicity, the accuracy of navigation switching, and the intuitiveness and consistency of the gestures required to use the mini program. The mean score is 3.881 (standard deviation = 0.762), which is the highest score among all sub scales.

The sub scale of aesthetics is used to assess comments on the mini program’s screen layout, graphic quality, visual appeal, and the consistency between color scheme and style. The mean score is 3.788 (standard deviation = 0.827).

The information quality scale evaluates the accuracy of the content of the mini program, the conciseness and briefness of information, the visual clearness and the credibility of the source. The mean score of this dimension is 3.936 (standard deviation = 0.731).

The subjective quality dimension asks for respondents’ overall opinion of the mini program, including their willingness to pay for the mini program, the frequency of using it, their score on the mini program, and whether they would like to recommend it to others. This dimension has a mean score of 3.171 (standard deviation = 0.945).

The perceived impact dimension evaluates the degree of influence, cognition, intention, and attitude and behavior change of the mini program on visiting Pingtan Tokai Wonderland. The mean score for this dimension is 3.956 (standard deviation = 0.783).

The results of Pearson correlation analysis in Table 7 show that the correlation coefficients between engagement, functionality, aesthetics, information quality, subjective quality and perceived impact range from 0.543 to 0.659, all reaching the significantly correlated level (p<0.05), which indicates that there is a significant positive correlation between engagement, functionality, aesthetics, information quality, subjective quality, and perceived impact. Therefore, further regression analysis will be performed to verify the impact of engagement, functionality, aesthetics, information quality and subjective quality on perceived impact.

**Table 7:** Results of Pearson correlation analysis.

	Engagement	Functionality	Aesthetics	Information quality	Subjective quality	Perceived impact
Engagement	1					
Functionality	.707**	1				
Aesthetics	.727**	.745**	1			
Information quality	.724**	.726**	.799**	1		
Subjective quality	.727**	.591**	.721**	.740**	1	
Perceived impact	.603**	.543**	.607**	.659**	.644**	1

** Significantly correlated at the .01 level (two-sided).

Engagement, functionality, aesthetics, information quality, and subjective quality are regarded as independent variables, and perceived impact dependent variable, the model was obtained through multiple linear regression analysis. It can be seen from Table 8 that the R-square of the model is 0.499, and the adjusted R-square is 0.487, indicating that the goodness of fit between the model and the data is relatively high.

**Table 8:** Summary of regression analysis model.

Model summary				
Model	R	R-square	Adjusted R-square	Standard Error of Estimate (SEE)
1	.707a	0.499	0.487	0.56074

a. predictable variable: (constant), subjective quality, functionality, engagement, information quality, aesthetics.

The F value of the Anova model test in Table 9 is 42.279, and the corresponding sig is close to 0 and less than 0.05, indicating that the regression coefficients of the independent variables are not 0 at the same time, and there is a significant linear correlation between at least one independent variable and the dependent variable in the regression model. Therefore, the choice of model is reasonable.

**Table 9:** Test results of Anova model.

Anova						
Model		Sum of square	df	Mean square	F	Sig.
1	Regression	66.468	5	13.294	42.279	.000b
	Residual	66.659	212	.314		
	Total	133.127	217			

a. dependent variable: perceived impact

b. predictable variable: (constant), subjective quality, functionality, engagement, information quality, aesthetics.

Table 10 shows the results of the regression coefficient significance test between each variable and the dependent variable. It can be seen from the table that the standardized regression coefficients of engagement, functionality and aesthetics on perceived impact are 0.121, 0.041 and 0.056 respectively, which are not significant correlation (p>0.05), indicating that engagement, functionality and aesthetics have no significant impact on perceived impact. The standardized regression coefficients of information quality and subjective quality on perceived impact are 0.295 and 0.274, which constitute significant correlation (p<0.05), indicating that information quality and subjective quality have a significant positive impact on perceived impact. The higher the information quality and subjective quality of the APP, the greater the perceived impact of the product on users. In addition, the standardized regression coefficient of information quality on perceived impact is greater than that of subjective quality on perceived impact, indicating that information quality has a greater impact on perceived impact. Therefore, the mini program should be strengthened in terms of information quality and APP development.

**Table 10:** Results of the regression coefficient significance test between each variable and the dependent variable.

Coefficient a						
Model		Unstandardized coefficient		Standardized coefficient	t	Sig.
		B	Standard error	Trial		
1	(constant)	1.223	.223		5.476	.000
	Engagement	.115	.080	.121	1.440	.151
	Functionality	.042	.083	.041	.502	.616
	Aesthetics	.053	.088	.056	.597	.551
	Information quality	.316	.100	.295	3.172	.002
	Subjective quality	.227	.068	.274	3.352	.001

a. dependent variable: perceived impact

## 5. Discussion

The practical design verifies that the technical presentation of tilt photography and the collection and presentation of Internet back-end data are carried by wechat mini program. The statistical results of user feedback and usability questionnaire test show that the basic function of Donghai Fairyland applet is normal and the interface is smooth. The overall internal consistency of MARS is very good, with Cronbach’s Al-pha value of 0.961. This result is similar to the original English version [35]. In addition, internal consistency is relatively good for engagement, functionality, aesthetics, information quality, application subjective quality, and perceived influence sub-scales (α = 0.852, 0.847, 0.838, 0.856, 0.837, and 0.939, respectively). Our results are similar to or superior to those of other validation studies for MARS [35, 39, 40]. The descriptive statistical results show that the largest dimension of the mean value is "perceived impact", indicating that experiential participants believe that the use of Donghai Fairyland mini program has positive behavioral changes for traveling to Pingtan, which is also the practical purpose of this study. Under the multi-complex development trend of digital marketing in the era of big data, it can play a role in the marketing and in-depth development of tourist destinations.

In addition, Pearson correlation analysis showed that participation, functionality, aesthetics, information quality, subjective quality of applications were significantly positively correlated with perceived impact. Further regression analysis shows that participation, functionality and aesthetics have no significant influence on perception. Information quality and application subjective quality have significant positive influence on perceived impact. This indicates that there is a significant correlation between these subitems and perceived impact, but there is no regression correlation between participation, functionality and aesthetics. That is, H1-H3 is not valid, and H4 and H5 hypotheses are valid. It shows that the entertainment, interface interaction, the layout and size of buttons, ICONS, menus and content on the screen and other aesthetics of the small program demo in the East China Sea Wonderland are not important factors that affect consumers’ decision to travel to the destination. In particular, the regression coefficient results show that the information quality has a greater impact on the perceived impact. This is consistent with feedback from users, most of whom want to enrich the details page. Showing that the content of the app is correct, organized, and clearly presents information about the destination, as well as the reliability of the source of the information, is what the public will be most concerned about. Therefore, the upgraded version of Donghai Fairyland mini program in the future should focus on strengthening the direction of information quality, especially in the direction of credibility and evidence base. In particular, the introduction of scenic spots in tourist destinations, the practicability of tour guides, transportation, accommodation, catering, accompanying gift and cultural creation products and other information are strengthened. In terms of visual information, the design is arranged in a graphic way, and the design is younger, in line with the popular style of the current young people.

## 6. Conclusion and limitations

### 6.1. Theoretical significance

This study explores the limitations of social media in travel marketing and the importance of oblique photography in enhancing the function of travel marketing. The innovation of this research lies in the successful realization of 3D real world imaging technology of tilt photography and its application to social media platforms from PC to mobile. Through wechat mini program as the carrier, the digital service is realized, which promotes the development of tourism and meets the needs of tourism participants. In addition, the research provides a more diversified and consistently effective service to tourism-related stakeholders through the carrier of limitless future possibilities. By combining oblique photography with social media and building small programs, the study innovates in data collection and presentation. By obtaining various data related to tourist destinations in the form of tourism services, this research can eventually provide services to the stakeholders related to tourist destinations. This study redefines the way of interaction between tourism destinations, tourism destination operators and tourists, provides new ideas and directions for the sustainable development of tourism destinations, and lays a theoretical and experimental foundation for further research and development.

### 6.2. Actual impacts

From the point of view of digital technology, the infinite possibilities of small program back-end development combined with the development of slanting photography backstage function, the continuous improvement of the technology called in the way of web pages, provides more forward-looking and predictive services for the future. By linking the information between tourists and each part of the consumption link of tourism, a new consumption ecology and consumption pattern can be formed. Therefore, we can build different modular designs for different travel destinations and choose different marketing tools and interaction methods. Wechat mini program provides navigation, tour guide, shopping guide sharing and public services, as well as real-time tourism promotion to help tourists reasonably plan their itinerary in advance and save decision-making time. Through the online payment function of the mini program, tourists can enjoy one-stop "inclusive" services, which can improve the satisfaction of tourists and promote the growth of tourism consumption and tourism economy.

The wechat mini program mentioned so far is just an example of an application carrier. The functional interaction of slant photography imaging data can be considered as a basic model. Slant photography can be combined with the application via a web call and presented to different users on different platforms as long as the application’s background port allows. The East China Sea Wonderland attractions mini program case can provide reference for other social media (such as Facebook, Instagram, LinkedIn, Line and Twitter, etc.) or travel and accommodation applications (such as popular Qunar travel, Feizhu, elong Travel, Ctrip, Hornet’s Nest, Tounu Travel, etc.). The combination of having extensive user statistics and the emerging technology of slanting photography enables users to interact with destinations online like never before. This model can be extended to different countries, different travel destinations and different applications, which needs further development and researchers to carry out practical application and verification.

### 6.3. Limitations and future research

By means of observation, interview and usability test questionnaire, this study obtained the future correction direction of small program, and verified the feasibility of mobile social media combined with tilt photography technology on the tourism online service platform. However, this study has the following limitations, which provide guidance for future research directions:

1. Usability test was only conducted in the first version of the small program successfully launched, and the destination information and interface design have not been fully incorporated into the practical design. In addition, the smooth HD presentation of panoramic imaging is affected by network bandwidth, but with the popularization of 5G, this problem will be solved. Future studies can be conducted with a wider range of users after the interface is corrected.
2. The incline photogrammetry technology involved in this study combined with the development of small programs has only realized the three-dimensional real scene presentation and the mounting of interest buoys in scenic spots. With the development of technology, in addition to meeting the needs of travel functions, the latest technology can also be combined, such as the local sound, environment or weather factors into the application interface, so as to achieve a more comprehensive and diversified destination reality presentation and experience. Future research needs to continue to focus on imaging technology and explore the combination of oblique photography and various advanced technologies in order to achieve greater value of oblique photography.
3. At present, there is no data precipitation in the background of mini program, so it is impossible to carry out targeted discussion on tourism decisions. Future studies can further analyze the precipitated data to verify the advantages of this new business path.

These limitations suggest the direction of future research. Optimizing the function and interface design of small programs, exploring the integration of oblique photography with other advanced technologies, and in-depth analysis of the precipitation data will help further enhance the effect and value of the tourism online service platform.

## Supporting information

S1 Dataset (XLSX)
